# Supplementary material for: A probiotic bi-functional peptidoglycan hydrolase sheds NOD2 ligands to regulate gut homeostasis in female mice
Source: Nat Commun. 2023 Jun 7;14:3338. doi: 10.1038/s41467-023-38950-3 (PMC10247697; doi:10.1038/s41467-023-38950-3)

A probiotic bi-functional peptidoglycan hydrolase sheds NOD2 ligands to regulate gut homeostasis in female mice

Jie Gao[1,ǂ], Lei Wang[1,ǂ], Jing Jiang[2,ǂ], Qian Xu[1,ǂ], Nianyi Zeng[1], Bingyun Lu[3], Peibo Yuan[1], Kai Sun[4*], Hongwei Zhou[1,5*], Xiaolong He[1*]

[1]Microbiome Medicine Center, Department of Laboratory Medicine, Zhujiang Hospital, Southern Medical University, Guangzhou, Guangdong, 510655, China.

[2]Department Gerontology, Sichuan Academy of Medical Sciences & Sichuan Provincial People's Hospital, Chengdu, Sichuan, 610072, China.

[3]Department of Gastroenterology, Shenzhen Hospital, Southern Medical University, Shenzhen, Guangdong, 518101, China.

[4]Department of General Surgery, Nanfang Hospital, Southern Medical University, Guangzhou, Guangdong, 510515, China.

[5]State Key Laboratory of Organ Failure Research, Southern Medical University, Guangzhou, Guangdong, 510655, China.

[ǂ]These authors contributed equally to this study

[*]Correspondence: biodegradation@gmail.com (H.Z.), hxl2027315@smu.edu.cn (X.H.) sunkai9602@sina.com (K.S.)

Supplementary Information

Supplementary Figure 1

Supplementary Figure 2

Supplementary Figure 3

Supplementary Figure 4

Supplementary Figure 5

Supplementary Figure 6

Uncropped scans of all gels in Supplementary Figure 3

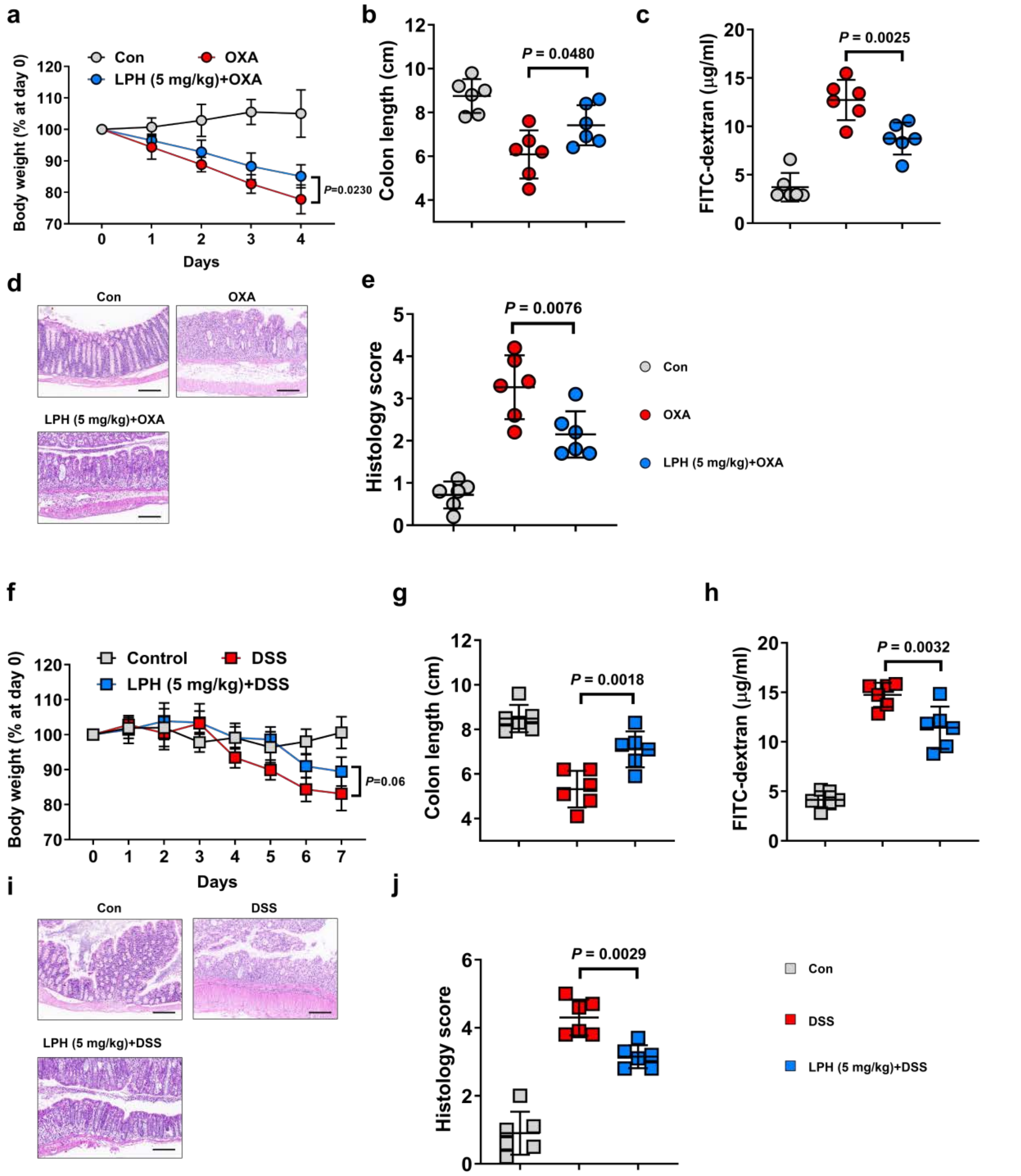

Supplementary Figure 1. LPH-treated mice were also resistant to OXA- or DSS-induced colitis. a, f Body weight changes of OXA- (**a**) or DSS-induced colitis model (**f**). Colon length (**b**, **g**); serum FITC-dextran level (**c**, **h**); representative H&E staining of colonic tissue, scale bar, 200 μm (**d**, **i**), and inflammation score (**e**, **j**). n = 6 mice per group, female. OXA: oxazolone; DSS: dextran sulfate sodium. Data are representative of 2 independent experiments and presented as mean ± SD. Statistical analyses were performed using repeated measures ANOVA with Bonferroni post hoc test (a, f), one-

way ANOVA with Bonferroni post hoc test (b, c, e, g, h, j). Source data are provided as a Source data file.

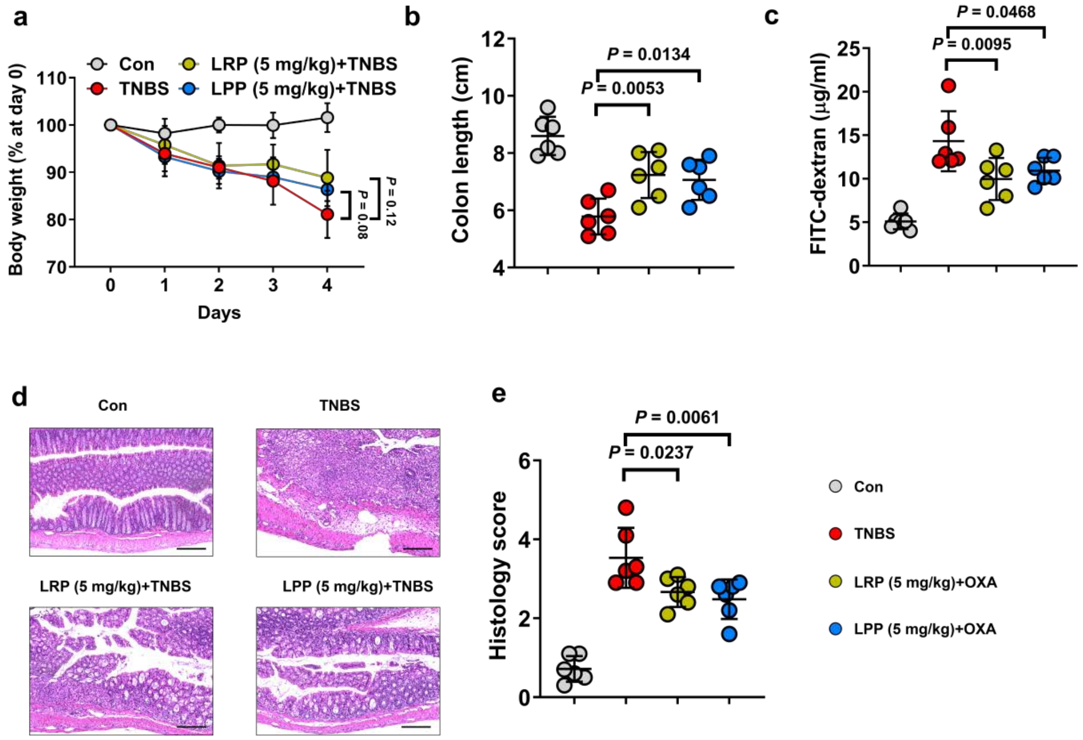

Supplementary Figure 2. Two homologues of LPH from *L. rhamnosus* and *L. paracasei* could also protect mice from TNBS-induced colitis. a-e Body weight loss (**a**); colon length (**b**); serum FITC-dextran level (**c**); representative H&E staining of colonic tissue, scale bar, 200 μm (**d**), and semiquantitative scoring of inflammation (**e**) of mice from indicated groups. LRP: homologues of LPH from *L. rhamnosus*; LPP: homologues of LPH from *L. paracasei*. Data are representative of 2 independent experiments and presented as mean ± SD. Each dot indicates an individual mouse (n = 6, female). Statistical analyses were performed using repeated measures ANOVA with Bonferroni post hoc test (a), one-way ANOVA with Bonferroni post hoc test (b, c, e). Source data are provided as a Source data file.

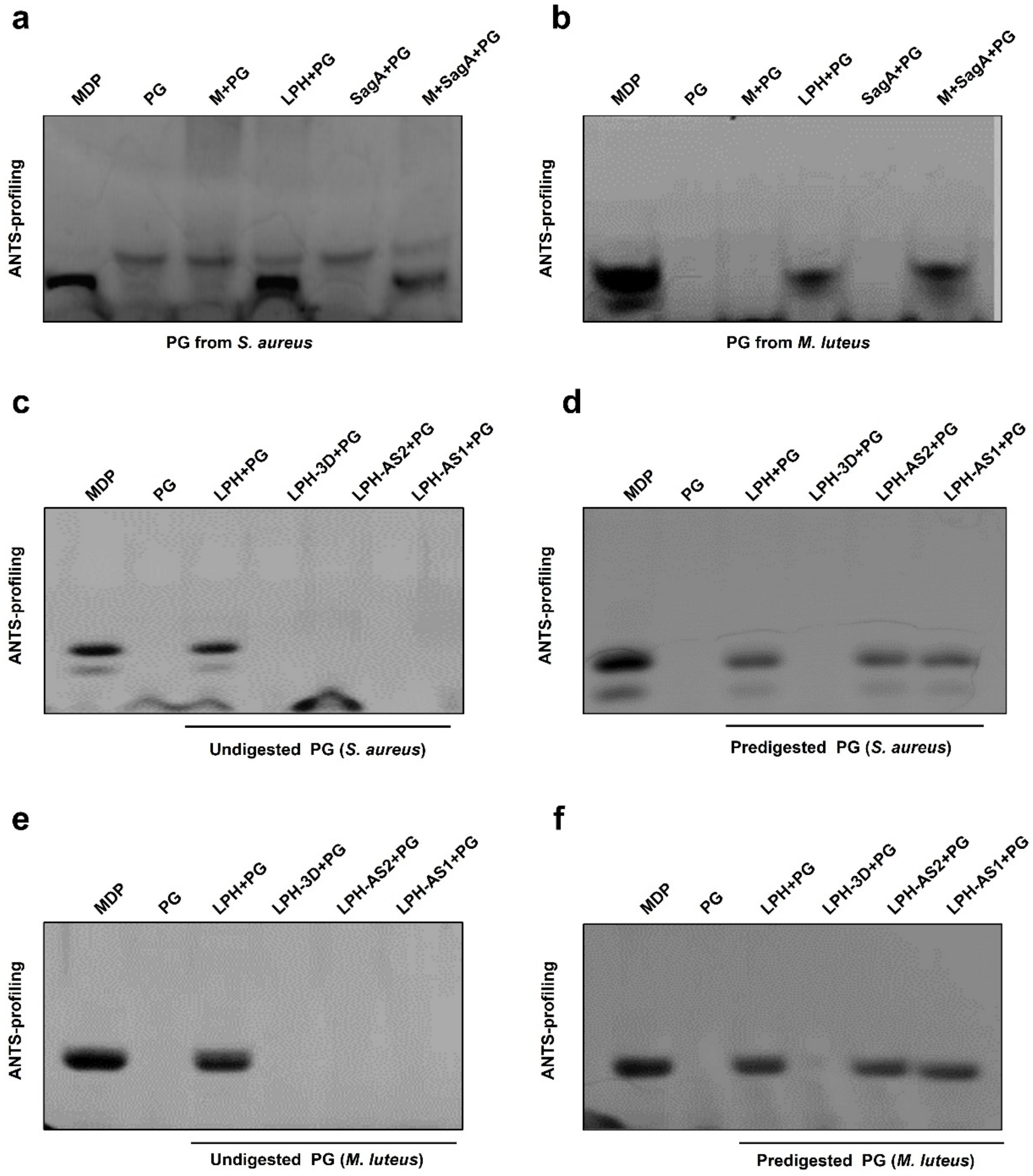

Supplementary Figure 3. Evaluation of LPH's peptidoglycan hydrolytic activity using ANTS labeled gel electrophoresis. a-b ANTS visualization of peptidoglycan from *S. aureus* (**a**) or *M. luteus* (**b**) digested with indicated enzymes suggested that LPH has a similar function with a combination of mutanolysin (M, N-Acetyl-β-D-muramidase) and SagA (DL-endopeptidase). **c-f** ANTS visualization of peptidoglycan from *S. aureus* (**c**, **d**) or *M. luteus* (**e**, **f**) digested with indicated enzymes suggested that LPH-AS1/LPH-AS2 lost the ability to generate MDP from intact peptidoglycan while preserving the ability to generate MDP from mutanolysin predigested peptidoglycan. Data are representative of two independent experiments. PG: peptidoglycan; LPH-AS1: D316A mutation of LPH; LPH-AS2: D329A mutation of LPH; LPH-AS3: P295A,

G297A, and T298A mutations of LPH; LPH-3D, the 3D-domain truncation of LPH; Mutanlolysin (M) was used as a positive control for N-Acetyl-β-D-muramidase; SagA was used as a positive control for DL-endopeptidase.

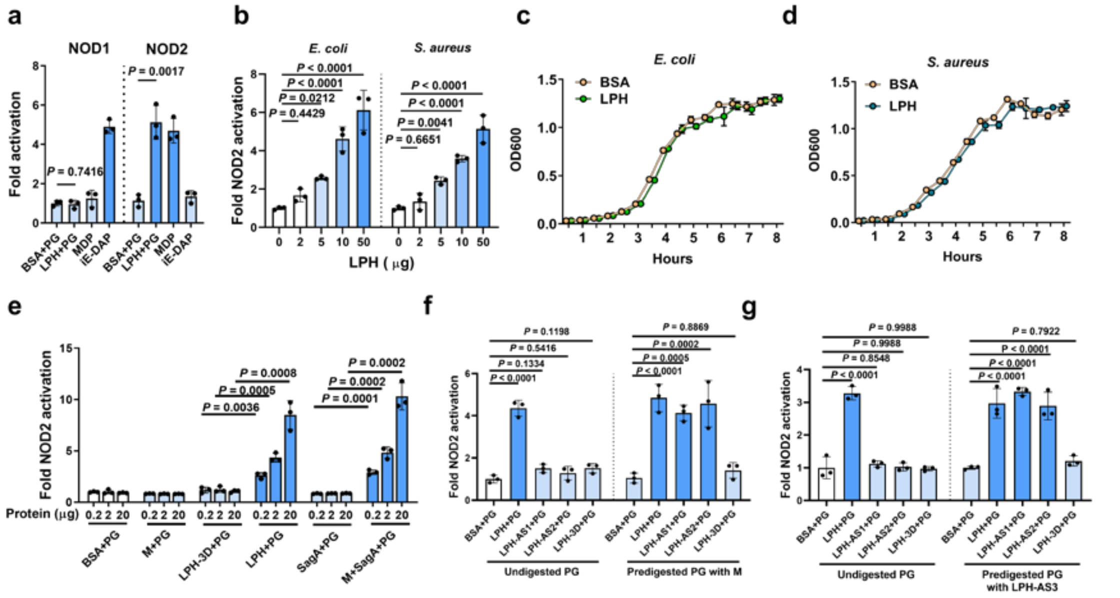

Supplementary Figure 4. Evaluation of LPH's peptidoglycan hydrolytic activity using HEK293 NOD reporter cells. a NF-κB activation in NOD1 and NOD2 reporter cells after incubation with BSA or LPH-treated peptidoglycan. NOD1 agonist (iE-DAP) and NOD2 agonist (MDP) served as the positive control. **b** Cell pellets of *E. coli* or *S. aureus* (2.5×10^7 colony forming unit) were digested with different doses of LPH as indicated, and the ability of digests to activate NOD2-expressing NF-κB reporter HEK293 cells were detected. n = 3 per group. **c-d** The growth curve of *E. coli* (**c**) or *S. aureus* (**d**) in a medium containing 20 μg/ml BSA or LPH. **e** NF-κB activation in NOD2 reporter cells after incubation with BSA-, M-, LPH-3D-, LPH-, SagA- or M+SagA-treated peptidoglycan. **f-g** Peptidoglycans were predigested with or without M (**f**)/LPH-AS3 (**g**), and incubation with BSA, LPH, LPH-AS1, LPH-AS2, or LPH-3D. The ability of peptidoglycan digests to activate NF-κB in NOD2 reporter cells were detected. BSA: bovine serum albumin; PG: peptidoglycan; LPH-AS1: D316A mutation of LPH; LPH-AS2: D329A mutation of LPH; LPH-AS3: P295A, G297A, and T298A mutations of LPH; LPH-3D, the 3D-domain truncation of LPH; Mutanlolysin (M) was used as a positive control for N-Acetyl-β-D-muramidase; SagA was used as a positive control for DL-endopeptidase. The values are ratios of cells with corresponding treatment to those with BSA treatment. Data are representative of 3 independent experiments and presented as mean ± SD. Each dot indicates an individual sample. Statistical analyses were performed using one-way ANOVA with Bonferroni post hoc test (a, b, f, g), two-tailed unpaired Student's t-test (e). Source data are provided as a Source data file.

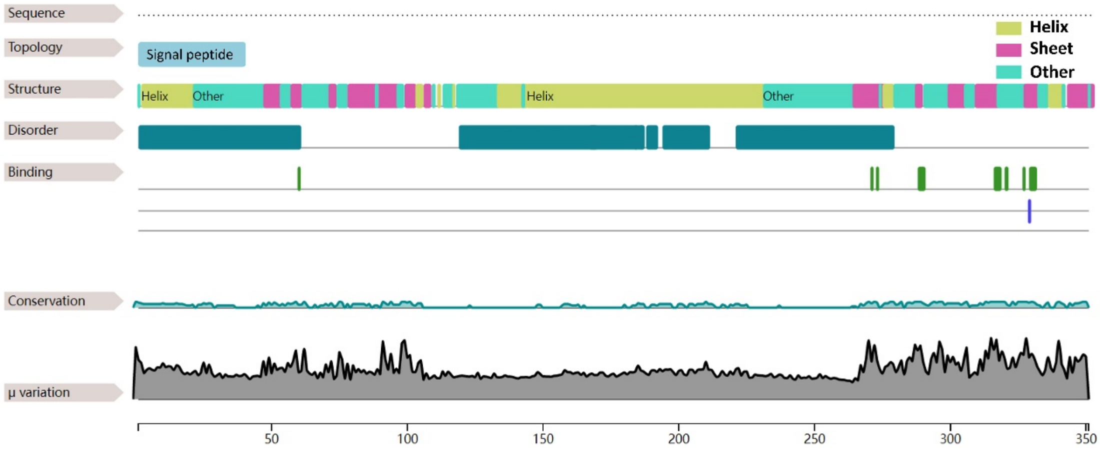

Supplementary Figure 5. The predicted secondary structure of LPH. The secondary structure of LPH was predicted using the web tool LambdaPP pipeline (https://embed.predictprotein.org/o). LPH has 40% α-helix and 20% β-sheet. The N-terminal (1-143) and C-terminal of LPH is composed of helix and sheet (231-351), while the middle of protein is mainly helix (144-230). Two conserved aspartic acids (D316, D329) were predicted as binding sites.

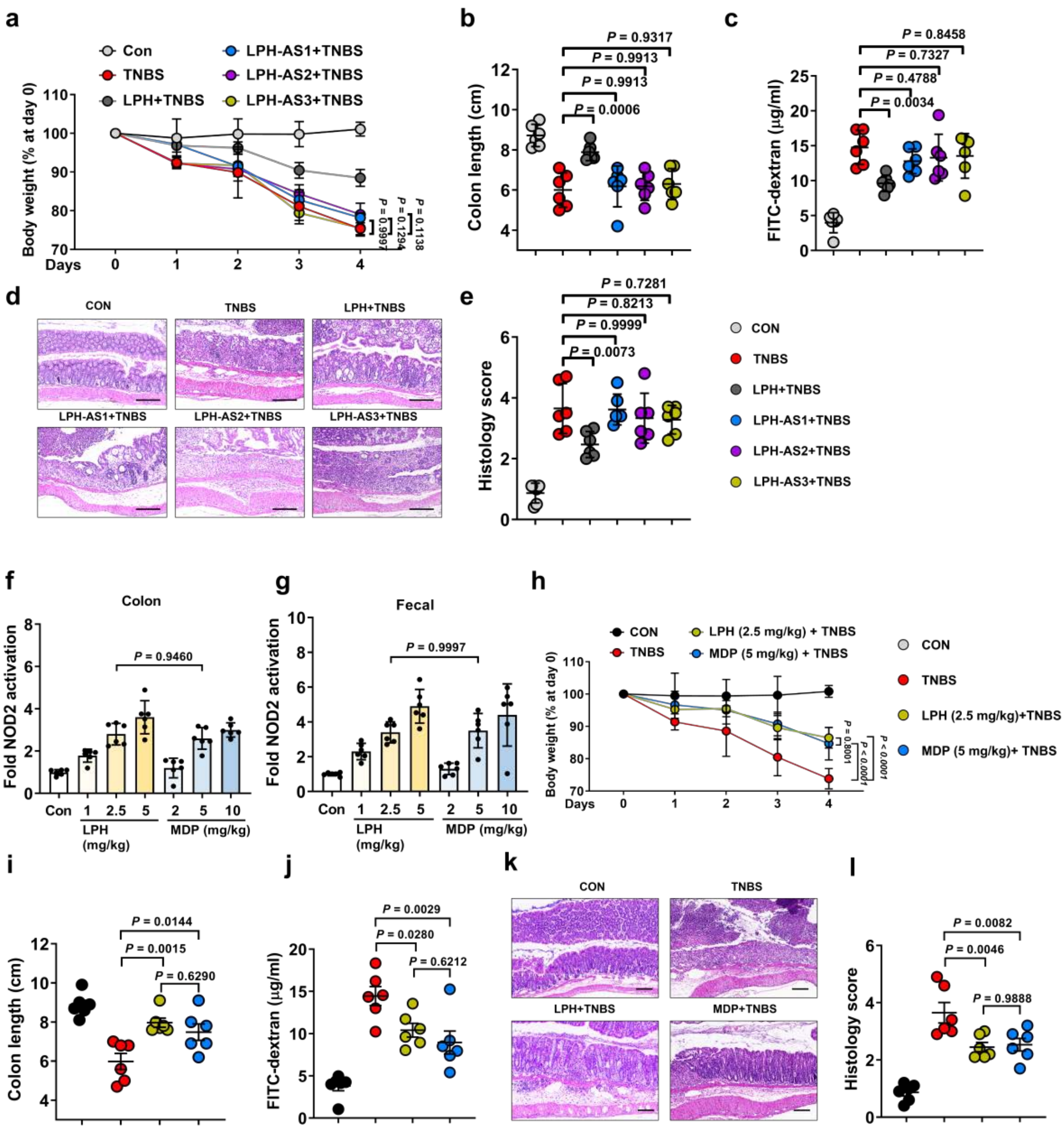

Supplementary Figure 6. The intact PGH activity is required and the generated MDP is sufficient for LPH's colitis-protective effects. a-e Mice were pretreated with pectin/zein beads containing LPH or its different active sites mutants at 1 mg/kg body weight for 3 days, followed by challenge with TNBS for 4 days. The colitis protective effects were evaluated: body weight loss (**a**), colon length (**b**), serum FITC-dextran level (**c**), representative H&E staining of colonic tissue, scale bar, 200 μm (**d**), and the semiquantitative scoring of inflammation (**e**). **f-g** Mice were gavaged with indicated dosage of pectin/zein beads containing LPH (1-5 mg/kg body weight) or MDP (2-10 mg/kg body weight), and the ability of colonic homogenate (**f**) or fecal extract (**g**) to activate NOD2-expressing NF-κB reporter HEK293 cells were detected. Data are presented as fold change relative to BSA treated group (control). **h-l** Mice were pre-

treated with pectin/zein beads containing LPH (2.5 mg/kg) or MDP (5 mg/kg) before being challenged with TNBS for 4 days. The protective effects of LPH and MDP on colitis were evaluated by measuring body weight loss (**h**), colon length (**i**), serum FITC-dextran level (**j**), and semiquantitative scoring of inflammation (**l**). Representative H&E staining of colonic tissue was also performed, and the scale bar is 200 μm (**k**). Each dot indicates an individual mouse (n = 6, female). Data are representative of 3 independent experiments and presented as mean ± SD. Statistical analyses were performed using repeated measures ANOVA with Bonferroni post hoc test (a, h), one-way ANOVA with Bonferroni post hoc test (b, c, e, i, j, l), two-tailed unpaired Student's t-test (f, g). Source data are provided as a Source data file.

Uncropped scans of all gels in Supplementary Figure 3.

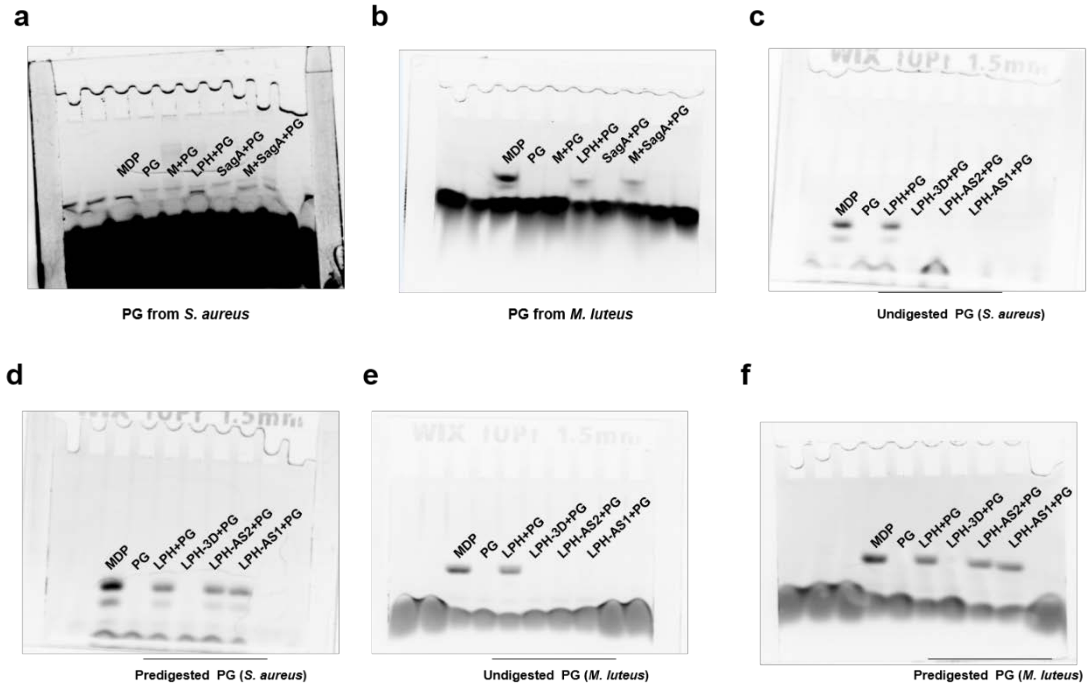

Supplement: Supplementary file 1 — Supplementary Information [file 41467_2023_38950_MOESM1_ESM.pdf]
