# Supplementary material for: A probiotic bi-functional peptidoglycan hydrolase sheds NOD2 ligands to regulate gut homeostasis in female mice
Source: Nat Commun. 2023 Jun 7;14:3338. doi: 10.1038/s41467-023-38950-3 (PMC10247697; doi:10.1038/s41467-023-38950-3)
Supplement: Supplementary file 3 — Description of Additional Supplementary Files [file 41467_2023_38950_MOESM3_ESM.pdf]

Description of Additional Supplementary Files

File Name: Supplementary Data 1
Description: Three hundred and sixty-four predicted secreted proteins from 10 Lactobacillus strains categorized into 159 clusters at 75% identity.

File Name: Supplementary Data 2
Description: The OTU abundance of individual mice in Figure 5.
