# Supplementary material for: A probiotic bi-functional peptidoglycan hydrolase sheds NOD2 ligands to regulate gut homeostasis in female mice
Source: Nat Commun. 2023 Jun 7;14:3338. doi: 10.1038/s41467-023-38950-3 (PMC10247697; doi:10.1038/s41467-023-38950-3)
Supplement: Supplementary file 6 — Reporting Summary [file 41467_2023_38950_MOESM6_ESM.pdf]

nature portfolio

Corresponding author(s): Xiaolong He, Hongwei Zhou, Kai Sun

Last updated by author(s): May 21, 2023

Reporting Summary

Nature Portfolio wishes to improve the reproducibility of the work that we publish. This form provides structure for consistency and transparency in reporting. For further information on Nature Portfolio policies, see our Editorial Policies and the Editorial Policy Checklist.

Statistics

For all statistical analyses, confirm that the following items are present in the figure legend, table legend, main text, or Methods section.

n/a	Confirmed	
☐	☒	The exact sample size (*n*) for each experimental group/condition, given as a discrete number and unit of measurement
☐	☒	A statement on whether measurements were taken from distinct samples or whether the same sample was measured repeatedly
☐	☒	The statistical test(s) used AND whether they are one- or two-sided<br>*Only common tests should be described solely by name; describe more complex techniques in the Methods section.*
☒	☐	A description of all covariates tested
☐	☒	A description of any assumptions or corrections, such as tests of normality and adjustment for multiple comparisons
☐	☒	A full description of the statistical parameters including central tendency (e.g. means) or other basic estimates (e.g. regression coefficient) AND variation (e.g. standard deviation) or associated estimates of uncertainty (e.g. confidence intervals)
☐	☒	For null hypothesis testing, the test statistic (e.g. *F*, *t*, *r*) with confidence intervals, effect sizes, degrees of freedom and *P* value noted<br>*Give P values as exact values whenever suitable.*
☒	☐	For Bayesian analysis, information on the choice of priors and Markov chain Monte Carlo settings
☒	☐	For hierarchical and complex designs, identification of the appropriate level for tests and full reporting of outcomes
☒	☐	Estimates of effect sizes (e.g. Cohen's *d*, Pearson's *r*), indicating how they were calculated

Our web collection on statistics for biologists contains articles on many of the points above.

Software and code

Policy information about availability of computer code

Data collection	PSORTb (v3.0); CD-hit (v4.8.1); PFAM (v35.0); Modeller (v9.22-1); PyMol (v2.4.0); AlphaFold (v2.0); SignalP (v5.1); NIS-Elements Basic Research Imaging Software (v3.2); Fastp (v0.20.0); FLASH (v1.2.7); UPARSE (v7.1); RDP Classifier (v2.2); Silva (v138); Thermo Xcalibur software (v4.0)
Data analysis	ImageJ (v1.49); GraphPad Prism v8.0.2

For manuscripts utilizing custom algorithms or software that are central to the research but not yet described in published literature, software must be made available to editors and reviewers. We strongly encourage code deposition in a community repository (e.g. GitHub). See the Nature Portfolio guidelines for submitting code & software for further information.

Data

Policy information about availability of data

All manuscripts must include a data availability statement. This statement should provide the following information, where applicable:

- Accession codes, unique identifiers, or web links for publicly available datasets
- A description of any restrictions on data availability
- For clinical datasets or third party data, please ensure that the statement adheres to our policy

The authors declare that all data supporting the findings of this study are available within this paper and its Supplementary Files. The Microbiome (16S rRNA sequencing) data generated in this study have been deposited into the Sequence Read Archive (SRA) in National Center for Biotechnology Information (NCBI) under the accession NO. PRJNA973702 (https://www.ncbi.nlm.nih.gov/sra/PRJNA973702). The Silva database used in 16S rRNA microbial analysis is download from

https://www.arb-silva.de/. The genomes of probiotics are downloaded from NCBI: https://www.ncbi.nlm.nih.gov/genome/. Source data are provided with this paper.

Human research participants

Policy information about studies involving human research participants and Sex and Gender in Research.

Reporting on sex and gender	N/A
Population characteristics	N/A
Recruitment	N/A
Ethics oversight	N/A

Note that full information on the approval of the study protocol must also be provided in the manuscript.

Field-specific reporting

Please select the one below that is the best fit for your research. If you are not sure, read the appropriate sections before making your selection.

☒ Life sciences ☐ Behavioural & social sciences ☐ Ecological, evolutionary & environmental sciences

For a reference copy of the document with all sections, see nature.com/documents/nr-reporting-summary-flat.pdf

Life sciences study design

All studies must disclose on these points even when the disclosure is negative.

Sample size	As this study was a exploratory, the sample size was not calculated. 5-7 mice were needed in each group based on our previous experiments performed in the lab using the same models. Sample size for in vitro study were based on years of experience using NOD1/NOD2 reporter cells.
Data exclusions	No data were excluded from the analyses.
Replication	Replication was performed in all experiments and samples when possible. In vitro/ in vivo experiments were replicated with similar results, and the specific number of experiments performed during this study is also provided in the Figure legends.
Randomization	Mice were randomly assigned to groups according to their similar bodyweight and age prior to TNBS/DSS/OXA or AOM/DSS treatment. For cell experiments, HEK-Blue™ hNOD1 and HEK-Blue™ hNOD2 cell lines were parallel seeded and randomly assigned to different treatments.
Blinding	Prior to beginning of each in vivo study, the investigators were blinded to group allocation. Investigators were blinded for in vivo assays and objective assessments.

Reporting for specific materials, systems and methods

We require information from authors about some types of materials, experimental systems and methods used in many studies. Here, indicate whether each material, system or method listed is relevant to your study. If you are not sure if a list item applies to your research, read the appropriate section before selecting a response.

Materials & experimental systems

n/a	Involved in the study	
☐	☒	Antibodies
☐	☒	Eukaryotic cell lines
☒	☐	Palaeontology and archaeology
☐	☒	Animals and other organisms
☒	☐	Clinical data
☒	☐	Dual use research of concern

Methods

n/a	Involved in the study	
☒	☐	ChIP-seq
☒	☐	Flow cytometry
☒	☐	MRI-based neuroimaging

Antibodies

Antibodies used	Rabbit polyclonal anti-LPH antibody for WB: 1:5000; goat anti-rabbit IgG antibody conjugated with horseradish peroxidase (1:5000, Proteintech, catalog number SA00001-2)
Validation	This antibody was generated using recombinant histidine-tagged LPH in our lab. New Zealand white rabbits (6-8 weeks, 2 kg body weight) were immunized through repeated intradermal injections of LPH (1:1 emulsified in Freund's adjuvant). After the final boost, blood was collected and serum was prepared. The polyclonal antibody was purified using Pierce™ Protein A IgG Purification Kit. The antibody was validated using WB for detecting recombinant LPH with a dilution at 1:40000.

Eukaryotic cell lines

Policy information about cell lines and Sex and Gender in Research

Cell line source(s)	Human NOD/NF-κB/SEAP reporter HEK293 cells, including HEK-Blue™ hNOD1 and HEK-Blue™ hNOD2 were purchased from InvivoGene (Cat# hkb-hnod1; hkb-hnod2).
Authentication	The cell lines used in this study were HEK-Blue™ hNOD1 and HEK-Blue™ hNOD2, which were purchased from InvivoGene (Cat# hkb-hnodl; hkb-hnod2). They are commercial cell lines used to detection NOD1/2 activation as described in many study (PMID: 27742759; PMID:27039337; PMID: 36049483; PMID:36002575).These cell lines are not list in the International Cell Line Authentication Committee (https://iclac.org/databases/cross-contaminations/).
Mycoplasma contamination	All cell lines tested negative for mycoplasma contamination
Commonly misidentified lines (See ICLAC register)	No commonly misidentified cell lines were used in this study

Animals and other research organisms

Policy information about studies involving animals; ARRIVE guidelines recommended for reporting animal research, and Sex and Gender in Research

Laboratory animals	Female New Zealand white rabbits (6-8 weeks, 2 kg body weight); Female C57BL/6 mice, 6-8 weeks old; Female Nod2 knockout and wild type mice, on the background C57BL/6 (6-8 weeks old).
Wild animals	No wild animals were used in this study.
Reporting on sex	We used female mice in colitis model, as they are common used in chemically induced intestinal inflammation in our lab, with robust and reproducible data. The anti-LPH polyclonal antibody was generated using female New Zealand white rabbits, as female is sensitive with lower doses of antigen and have higher response to immunization than males. Male and female animals will be included in our future studies.
Field-collected samples	No samples collected from the field
Ethics oversight	The ethics committee of Southern Medical University approved the animal protocols and animal care and experiments were done strictly in adherence to the Southern Medical University animal care guidelines.

Note that full information on the approval of the study protocol must also be provided in the manuscript.
